# Changes in blink reflex after simultaneous supraorbital and mental nerve stimulations in healthy subjects

**DOI:** 10.55730/1300-0144.5823

**Published:** 2024-02-12

**Authors:** Ayşegül GÜNDÜZ, Tuba CERRAHOĞLU ŞİRİN, Pınar BEKDİK ŞİRİNOCAK, Tuba AKINCI, Burcu Nuran ARKALI, Fatma CANDAN, Meral E. KIZILTAN

**Affiliations:** Department of Neurology, Cerrahpaşa Medical Faculty, İstanbul University-Cerrahpaşa, İstanbul, Turkiye

**Keywords:** Simultaneous stimulation, trigeminal nerve, blink reflex, mental nerve, supraorbital nerve, recovery curve

## Abstract

**Background/aim:**

In this study, we investigated the blink reflex (BR) after simultaneous and asynchronous stimulation of two trigeminal nerve branches. The objective was to characterize the physiology of trigeminal and facial circuits.

**Materials and methods:**

We performed three sets of experiments: recording BR response i. after supraorbital nerve stimulation (SON), after mental nerve stimulation (MN), and after simultaneous SON and MN stimulation (SON+MN) in 18 healthy individuals; ii. after MN (at an intensity eliciting BR response) preceding SON at various interstimulus intervals (ISIs) in seven healthy subjects; iii after MN (at sensory threshold) preceding SON at various ISIs. We compared the magnitudes of early and late responses.

**Results:**

The R1 amplitude after simultaneous SON+MN stimulation was greater than responses after single stimulation of the same branches. After simultaneous stimulations, the R2 and R2c areas under the curve (AUC) were smaller than the arithmetic sums of R2 and R2c AUC obtained after single stimulations. The second experiment provided a recovery excitability curve. In the third step, we obtained facilitation of R1 and inhibition of late responses.

**Conclusion:**

The SON+MN stimulation caused an increased R1 circuit excitability compared to the arithmetic sum of the single stimulations; however, magnitudes of late responses did not potentiate. Thus, we have provided evidence for R1 circuit enhancement by simultaneous stimulation in humans, whereas modulation of late responses exhibited a recovery curve similar to that shown for paired SON stimulation.

## 1. Introduction

The blink reflex (BR) is the reflexive closure of the eyelids in response to any kind of stimulus. In routine electrophysiology practice, BR is usually obtained by the electrical stimulation of the supraorbital nerve (SON). This response consists of two components: (i) an ipsilateral R1 response (oligosynaptic) with a latency of 9–12 ms and (ii) bilateral polyphasic R2 responses (polysynaptic) with a latency of 25–35 ms [1,2]. The R1 response is generated at the pontine component of the trigeminal nucleus, whereas the R2 response originates within the ponto-medullary connections [3]. Paired supraorbital nerve stimulation at shorter intervals leads to the inhibition of the R2 response. As the interval increases, the output recovers [4]. The recovery excitability of BR provides information regarding the excitability of the BR pathway. The BR is also modulated by peripheral sensory stimulation, called the prepulse inhibition (PPI) of BR, and it reflects the filtering capacity of the brainstem interneurons [5].

Stimulating any branch of the trigeminal nerve may trigger R1 and R2 responses. However, the effect of stimulating different trigeminal nerve branches on BR is unknown. In this study, we investigated the BR response obtained by simultaneous and asynchronous stimulation of two trigeminal nerve branches. The objective was to characterize the physiology of trigemino-facial circuit further.

For this purpose, we chose SON and mental nerve (MN) stimulations because SON provides the largest and a rather stable BR response [6], and MN stimulation leads to slightly delayed and relatively smaller responses compared to those obtained after stimulation of other branches of the trigeminal nerve [6–9]. Since we used surface electrodes, we did not choose infraorbital nerve stimulation ( ION) stimulation in humans to avoid volume conduction. We also added additional control experiments to compare the present protocol to the conventional ones.

## 2. Subjects and method

### 2.1. Subjects

We performed three sets of experiments for SON and MN stimulations: i. single stimulations vs. simultaneous paired-pulse stimulation; ii. paired-pulse stimulation of MN (conditioning stimulus) and SON (test stimulus) at various interstimulus intervals (ISIs); and iii. prepulse stimulation (MN as the prepulse stimulus and SON as the test stimulus). In the first experiment, we included 18 healthy individuals (mean age: 37.3 ± 6.8 years; range: 27–48 years; 12 women, 66.7%) after giving their written informed consent. In the second and third experiments, seven healthy subjects participated in the study (mean age: 36.6 ± 6.4 years; range: 29–46 years; 3 women, 50%). The subjects enrolled in experiments 2 and 3 also participated in experiment 1. We performed conventional recordings (recovery excitability of BR and PPI of BR) in the 18 subjects who participated in the first experiment. A healthy individual was defined as someone who is not on regular medication and has no chronic diseases. Individuals with neurological and other systemic chronic diseases and those with regular drug use were not included in the study. None of the participants had a history of peripheral facial palsy or botulinum toxin injection into facial nerve-innervated muscles.

The review board of Istanbul University-Cerrahpasa, Cerrahpasa Medical Faculty approved the study. All methods were carried out according to relevant guidelines and regulations.

### 2.2. Method

All subjects were fully awake and relaxed during the experimental procedures. They all had a full night’s sleep before the examination and were seated in a quiet room. All the recordings were done using a Neuropack Sigma MEB-5504K, Nihon Kohden Medical, Tokyo, Japan device. We placed the surface recording electrodes on the orbicularis oculi (O.oc) muscle, the active electrode on the lower eyelid, the reference electrode on the outer eye corner, and the ground electrode on the forehead. Recordings were bilateral, whereas the stimulation was unilateral (right-sided). All stimuli were repeated five times, and the raw recordings were rectified and averaged. Responses with amplitudes greater than 50 μV were accepted as reflex responses.

#### 2.2.1. First experiment

**Figure 1:**
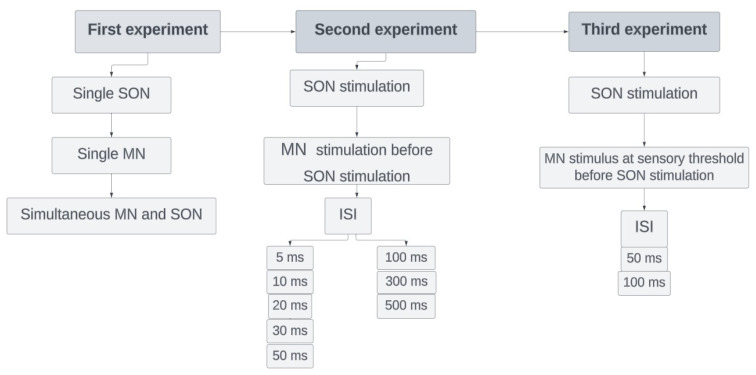
The diagram of our three experiments for recording blink reflex response. ISIs were randomly changed to avoid habituation.

In the first experiment, we recorded BR after the SON stimulation (single SON), after the MN stimulation (single MN), and after the simultaneous stimulation of the SON and MN (simultaneous MN and SON, SON+MN stimulation) (Figure 1). The electrical stimulation was given at the mouth corner for the MN stimulation and the supraorbital notch for the SON stimulation using a bipolar surface stimulator. A single 0.2-ms electrical stimulation was applied. The stimulus intensity was approximately four times the R2 threshold of SON and MN, respectively. All consecutive stimulations for a given nerve were at the same intensity in each individual. The filters were set to 20 and 2000 Hz. R1 and R2 or R2c were classified according to latency. To avoid habituation, there were at least 30 seconds between consecutive recordings.

#### 2.2.2. Second experiment

We recorded BR after the SON stimulation (test only), and we applied MN stimulation (conditioning stimulus) 5, 10, 20, 30, 50, 100, 300, and 500 ms before the SON stimulation (test stimulus) (Figure 1). The intensities of both conditioning and test stimuli were four times the R2 threshold of SON and MN, respectively. Again, we waited at least 30 s between consecutive recordings to avoid habituation, and ISIs were randomly changed.

#### 2.2.3. Third experiment

We recorded BR after the SON stimulation (test only), and we recorded BR response after the MN stimulus at sensory threshold 50 and 100 ms interstimulus intervals (ISIs) before SON stimulation (test stimulus) (Figure 1). The ISIs were chosen based on the previously published evidence of prepulse studies [14,25]. To avoid habituation, there were at least 30 seconds between consecutive recordings.

#### 2.2.4. Conventional experiments

We recorded the recovery excitability of BR and prepulse inhibition of BR. The recovery excitability of BR was recorded using 300- and 500-ms ISIs. The electrical stimulations for conditioning and test stimuli were delivered on the supraorbital notch using a bipolar surface stimulator. A paired, 0.2-ms electrical stimulation was applied. The stimulus intensity was approximately four times the R2 threshold of SON. All stimuli were repeated five times, and the raw recordings were rectified. We also recorded BR response after prepulse stimulus + SON stimulus (test stimulus) at 50 and 100 ms ISIs. As a prepulse stimulus, we used stimulation of the second finger at the sensory threshold.

## 3. Data and statistical analysis

No recordings were discarded. We measured the following parameters:

- For R1, onset latency and peak-to-peak amplitude in raw recordings,
- For R2 and R2c, onset latency and area under the curve (AUC) in rectified and averaged recordings.

The mean ± SD was calculated.

- In the first experiment, we calculated the arithmetic sum of R2 and R2c AUC values after the single MN and SON stimulations.
- In the second experiment, we calculated the percentage recovery (RC%) using the following formula offline: R2 or R2c AUC after test stimulus / R2 or R2c AUC after conditioning stimulus × 100.
- In the third experiment, we calculated the percentage of prepulse modulation (PPM%) using the following formula offline: R2 or R2c AUC after prepulse and test stimuli / R2 or R2c AUC after test only stimulus.

Onset latency was defined as the duration from the stimulus artifact to the beginning of the R1 or R2 responses. The average values were determined according to previous reports and established values from our laboratory [1,13].

When performing statistical analysis, we accepted amplitudes or AUC values as ‘0’ when there was no response. We did not consider it for latencies.

In the first experiment, we performed the Shapiro–Wilk test to analyze the normality of the data. We compared the values obtained after single SON, single MN, or SON+MN stimulations using the Wilcoxon signed-rank test or repeated measures ANOVA in binary comparisons. We also compared the mean R1 amplitudes as well as R2 and R2c AUC obtained after the simultaneous stimulations of SON+MN with the arithmetic sum of R2 and R2c AUC values after the single MN and the single SON stimulations using the Wilcoxon signed-rank test because of the nonnormal distribution of data.

In the second experiment, we compared the latency and amplitude of R1 and latency and AUC of R2 and R2c obtained after test-only stimulus with those obtained after conditioning and test stimuli using the Wilcoxon signed-rank test. We also performed the repeated measures ANOVA because multiple measures of the same variable were taken on the same subjects under different ISIs and post hoc tests for R2 and R2c. The factors in the model were various ISIs between conditioning and test stimuli from 0 up to 500 ms. Using RC%, we plotted the recovery curves.

In the third experiment, we compared the latency and amplitude of R1 and latency and AUC of R2 obtained after test-only stimulus with those obtained after prepulse and test stimuli using the Wilcoxon signed-rank test because of the nonnormal distribution of the data.

In the conventional experiments, we compared the latency and amplitude of R1 and latency and AUC of R2 obtained after test-only stimulus with those obtained after prepulse and test stimuli or conditioning and test stimuli using the Wilcoxon signed-rank test.

We also compared PPI or recovery excitability after paired stimulation of SON and stimulation of SON and MN using repeated measures ANOVA. The factors were subjects and different ISIs.

We calculated the delta difference between single stimulations and simultaneous stimulation. We analyzed the correlation between the change in R1 amplitude and the change in R2 AUC using the Spearman correlation test because of the nonnormal distribution of the data.

For statistical tests, SPSS package program version 20.0 (IBM, Armonk, NY, USA) was used, and p < 0.05 was deemed significant. The data were given as mean ± SD.

## 4. Results

### 4.1. First experiment

Early (R1) and late (R2 and R2c or contralateral R2) responses were recorded in all healthy subjects after each type of stimulation except the MN stimulation, after which the R1 response was recorded in seven (38.8%) individuals. The values obtained after the SON stimulation were normal according to the previously published values and the normal standards of our laboratory [1,10].

**Figure 2:**
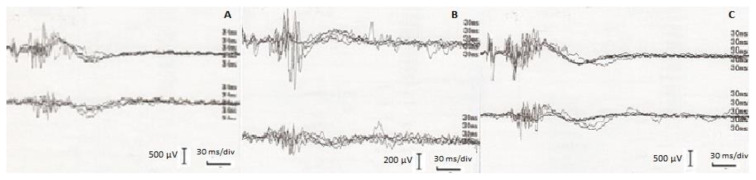
Blink reflex recordings after supraorbital (A), mental (B), and supraorbital+mental (C) nerve stimulations. The sensitivity change in column B because of the low-amplitude response should be noted.

R1 response after a single SON stimulation was larger than the R1 response obtained after a single MN stimulation (Table 1, Figures 2A–2C). Simultaneous SON+MN stimulation generated R1 responses with higher amplitudes than single stimulations (Table 1). R1 latency after SON+MN stimulation was shorter than the SON or MN stimulation (p = 0.003, F = 13.000 and p = 0.028, F = 8.346, respectively). The mean amplitude of R1 responses after simultaneous SON+MN stimulation was also more prominent than the arithmetic sum of the single stimulations in each individual.

The latencies of R2 and R2c responses after simultaneous SON+MN stimulation were significantly shorter compared to the responses elicited by the single stimulations (p = 0.000, F = 20.249 for SON+MN vs. SON, and p = 0.001, F = 16.811 for SON+MN vs. MN, Table 1). The magnitudes of R2 and R2c after simultaneous stimulation were significantly larger than the responses obtained after a single MN stimulation. In contrast, they were similar to those obtained after a single SON stimulation. Comparisons of the simultaneous SON+MN stimulation with the arithmetic sum of the values obtained after the single MN and single SON stimulations showed that R2 and R2c AUC in the respective recordings after the simultaneous SON+MN stimulation was significantly smaller (Table 2, p < 0.001 and p = 0.008, respectively).

### 4.2. Second experiment

No change in the R1 amplitude and latency was obtained after test-only stimulus and conditioning (MN) plus test (SON) stimuli at any ISI.

**Figure 3:**
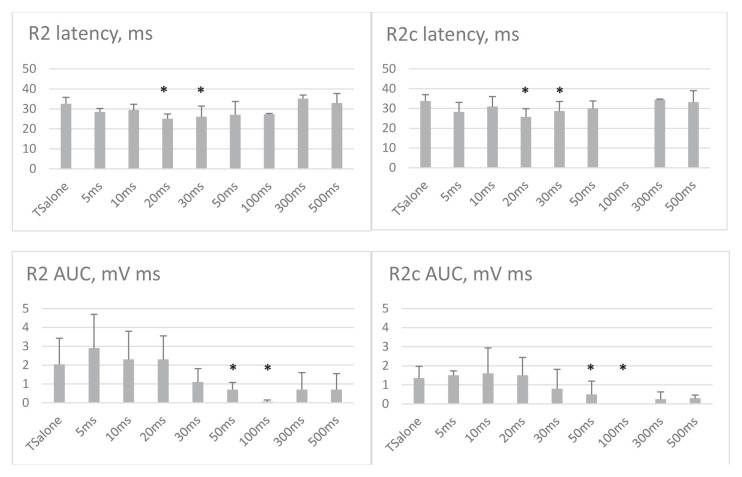
Changes of latency and area under the curve (AUC) of late responses at each ISI. The data are presented as mean ± SD. The R2c response is not elicited at all at 100 ms ISI. ‘*’ denotes significance at the < 0.05 level. X-axis denoted interstimulus intervals.

**Figure 4:**
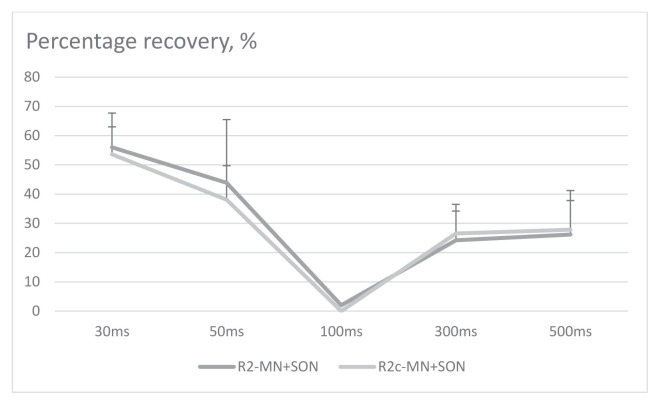
Percentage recovery of late responses after paired SON+MN stimulations at each interstimulus interval from 30 ms to 500 ms. The data are represented as mean ± SD. X-axis denoted interstimulus intervals.

Up until 30 ms ISIs, the R2 responses to the conditioning (MN) and test (SON) stimulations overlapped. Therefore, the responses seemed greater (Figure 3). The R2 magnitude was significantly reduced at 50 and 100 ms ISIs (p = 0.004, F = 16.471, df = 1 repeated measures ANOVA, post hoc analysis revealed p = 0.028; Figure 3). At 300 and 500 ms, R2 AUC was not significantly lower (Figure 3). R2c AUC was also lower at 50 and 100 ms ISIs (p = 0.005, F = 18.948, df = 1, post hoc analysis p = 0.038 repeated measures ANOVA). Figure 4 illustrates the recovery curves of late responses at each ISI.

The latency of the R2 response was significantly shorter when the conditioning stimulus was applied at 20 and 30 ms before the test stimulus compared to the test stimulus alone (p = 0.018 and p = 0.028, respectively). R2c latency was also shorter at 20 ms and 30 ms ISIs (p = 0.018 and p = 0.043, respectively), and at longer ISIs, the R2 and R2c latency was similar to that obtained by the test stimulus alone.

The R2 response obtained after conditioning plus test stimuli at each ISI was smaller than the arithmetic sum of the values obtained after the single MN and single SON stimulations.

### 4.3. Third experiment

**Figure 5:**
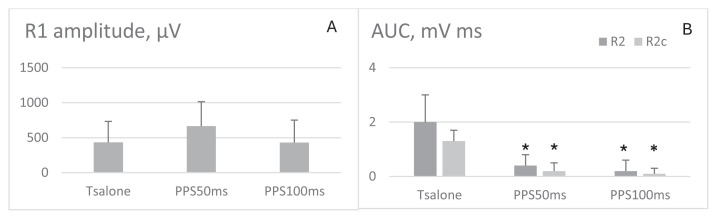
**A**. R1 amplitude after prepulse stimulation of mentalis nerve; **B**. R2 and R2c AUC after prepulse stimulation of mentalis nerve (Tsalone, test stimulus only condition; PPS, prepulse stimulation of MN and test stimulation of SON; AUC, area under the curve). The data are represented as mean ± SD. ‘*’ indicates significance level < 0.05 X-axis denoted interstimulus intervals.

There was a trend of higher R1 amplitude at 50 ms after the prepulse stimulation of MN (Figure 5A). There was no significant change of R1 at 100 ms ISI, whereas prepulse stimulation of MN effectively reduced the R2 and R2c magnitude at both ISIs (p = 0.008 for each, Figure 5B). There was no correlation between delta changes of R1 amplitude and R2 amplitude or AUC.

### 4.4. Conventional experiments

#### Recovery excitability of BR

The mean percentage recovery of R2 and R2c were 12% and 18%, respectively. The value reached 35% for both at 500 ms ISI. The values were quite similar to the respective ISIs of SON+MN stimulations.

#### PPI of BR

The R1 amplitude at 50 ms after the prepulse stimulation was higher, whereas there was no significant change of R1 at 100 ms ISI. The prepulse stimulation of the second finger provided inhibition of R2 and R2c. The values were similar to the respective ISIs of SON+MN stimulations.

The recovery excitability after paired stimulation of SON and stimulation of SON and MN were similar between subjects and different ISIs (for R2, p = 0.712, 0.515, df = 3.0 and for R2c, p = 0.632, F = 0.895, df = 3.0).

The PPI after stimulation of SON and second finger, as well as stimulation of SON and MN, were similar between subjects and different ISIs (for 50 ms ISI, p = 0.056, F = 17.061, df = 3.0 and for 100 ms, p = 0.107, F = 8.524, df = 3).

## 5. Discussion

The major findings of our study are as follows: i. The R1 amplitude increased after the simultaneous SON+MN stimulation compared to SON-only or MN-only stimulations, ii. R2 and R2c AUCs after simultaneous SON+MN stimulation were smaller than the arithmetic sum of R2 and R2c AUC obtained after the single stimulations, iii. A conditioning stimulus applied on MN reduced the R2 and R2c magnitude from 30 to 500 ms ISIs, while the prepulse stimulation of MN provided facilitation of R1 and inhibition of R2 and R2c. The recovery excitability and PPI obtained by MN stimulation were similar to those obtained by conventional methods.

Our results regarding the BR after the single MN stimulation are consistent with those of the previous studies [8,11]. In brief, the latencies were longer, and the stimulation thresholds needed to evoke a reflex response after the single MN stimulation were higher in previous studies. Similarly, we obtained the R2 and the R2c responses after the single MN stimulation with longer latencies and lower magnitudes relative to those obtained by SON stimulation. R1 after the single MN stimulation was more inconsistent, and we obtained it in only seven individuals. The R1 component similar to that obtained with the trigeminal V1 stimulation was rarely seen when simulating trigeminal V2, and it was never observed after trigeminal V3 stimulation [12]. A previous study suggested that the inputs from the MN easily habituate compared to the SON [9]. We took measures to avoid habituation. Therefore, in our opinion, inputs from MN were not strong enough to trigger an action potential in each case. Regarding the late responses after the MN stimulation, our findings supported the conclusion that they were repeatable and reliable; however, inputs from MN could trigger fewer O.oc motoneurons, i.e., weaker than SON stimulations.

### The effects on R1 circuitry

After simultaneous stimulation in experiment 1, the weaker MN inputs arrived during the SON stimulation. They enhanced the oligosynaptic R1 response produced by SON stimulation, suggesting the facilitation of its circuitry. However, there was no more effect on the R1 circuit with increasing ISIs starting from 5 ms, probably because the two inputs no longer coincide.

Low-intensity stimulation of MN provided facilitation of R1 at 50 ms, whereas there was no effect of similar intensity conditioning stimulus at 50 ms ISI. Therefore, the effect of low-intensity stimulus is probably through the prepulse facilitation of circuitry and sensory filtering at the brainstem since it was similar to that reported previously [14].

### The effects on R2 circuitry

Regarding late responses, during simultaneous SON+MN stimulation and SON+MN stimulation until 30 ms ISI, there was an overlap of the responses obtained after MN and SON stimulations. Despite the overlap, the magnitudes of the responses after simultaneous stimulation and SON+MN stimulations after an ISI (in experiments 1 and 2) were smaller than the arithmetic sum of the responses elicited by a single SON or single MN, suggesting an inhibition in the pathway. The inhibition was evident at 50 and 100 ms ISI, where the response was smaller than after the test-only stimulus. Many spinal sensory trigeminal neurons appear to respond only to the stimulation of a single trigeminal branch [15], suggesting a somatotopic organization in the sensory nucleus. In a healthy human, there is also a somatotopic functional architecture in the facial motoneurons [16]. Therefore, the lower R2 magnitude compared to the arithmetic sum should not directly relate to the sensory or motor pathway. When a paired-pulse stimulation at a certain ISI is given, the net effect of the test stimulus is reduced during the refractory period of the neurons producing the final behavior, and it is increased when the test stimulus arrives outside the refractory period [17]. The refractory period of facial motoneurons is around 2–3 ms [18]. The periods used in our study were beyond the refractory period. In the original study of BR recovery by Kimura and Harada [4], the authors showed that the R2 component was inhibited after paired stimulation of SON starting from the 40 ms ISI where the overlap of the two stimuli ended, reached a nadir at 80–140 ms and began to increase in magnitude from that time point until 800–1000 ms. We suggest that paired MN and SON stimulations created a similar recovery curve of R2 response. The authors attributed the changes of R2 to the functions of the interneuron pool, and subsequent studies suggested it was related to the excitatory mechanisms at the brainstem [19]. However, other possible mechanisms would explain the results of experiment 2 in our study. Innocuous stimuli can trigger both R1 and R2 [20]. R2 is mediated by medullary wide dynamic range neurons [11,20]. For example, noxious stimuli to the forearm were able to suppress R2 in previous studies [20]. The R2 inhibition in this study may be due to the convergence of innocuous SON and MN stimuli in the medullary wide dynamic range neurons rather than showing short-term plasticity changes at the brainstem BR circuit.

The results regarding late responses in experiments 1 and 2 may only point to a collision in the brainstem circuits. In physics, a collision is when two bodies suddenly and forcefully come into direct contact. When two bodies collide, the sum of the momenta of the bodies before impact is equal to the sum of the momenta after the impact. In electrophysiology, two similar types or different types of stimuli may collide, and the result will change the ultimate behavior [17]. However, theoretically, a collision may occur after simultaneous stimulation. This effect may continue until the overlap of the two stimuli continues, and this hypothesis cannot elucidate the inhibition after 30 ms ISI in experiment 2. However, we should carefully interpret the findings in the experiments 2 and 3 because of the small number of subjects.

A possible explanation for the results of experiment 1 is the “surround inhibition” (SI) present at multiple levels of the somatosensory system. In normal individuals, the sum of the two individual peripheral inputs is larger than the size of a dual input. SI is the suppression of the excitability of the area surrounding the active neural network. SI prevents unwanted movements in the motor system, increasing perception by enhancing contrast in the sensory system [21]. We can assume that the spinal trigeminal nucleus, which perceives ipsilateral facial touch and is the origin of component R2 of the BR, uses SI as a protective mechanism after a dual stimulus. However, we do not think SI is the putative mechanism explaining all findings regarding R2 in our study. Because SI has been shown to operate during simultaneous stimulations, the inhibition in longer ISIs in experiment 2 is probably not governed by SI.

Considering the different behavior of R1 and R2 responses, one such possible mechanism may be the sensory gating and filtering that occurs at the brainstem since we know that these two responses behave differently after the prepulse stimuli on different parts of the body [14]. A prepulse stimulus to MN also created a similar inhibition at 50 and 100 ms ISIs. However, prepulse stimulus means a stimulus that generates no reflex response [14]. Therefore, the conditioning response we used in experiment 2 should not be relevant in the prepulse circuit. For example, a low-intensity stimulus to MN in experiment 3 behaved like a prepulse stimulus.

There were certain limitations of this study. As we mentioned, there were seven participants in experiments 2 and 3. Thus, there is a likelihood of type II errors. In the third experiment, only two ISIs were tested based on previous studies. However, it could be interesting to test the ISIs used in experiment 2 to test the effects of conditioning stimulus at different intensities. The menstrual cycle of female participants was not noted. We know that studies are reporting the effect of birth control pills on spontaneous blink rates [22]. There are studies about the effect of progesterone on attentional blink [23] or the effect of psychotic episodes related to menstrual cycles on spontaneous blink rates [24]. Because of the close placement of the recording and stimulating electrodes, there were artifacts in recordings after ION stimulation. Therefore, we chose MN stimulation to avoid artifacts and propagation of the stimulus. One-sided examinations were performed based on the assumption that the participants were healthy individuals; therefore, there would be no difference between right and left-sided recordings because we avoided long examination sessions since we evaluated the various aspects of physiological changes in the trigemino-facial circuit and examination of both sides would double the recording time. The long duration of reflex examinations leads to changes in the parameters due to the characteristics of this cycle, such as habituation, or may affect cognitive functions such as attention, causing changes in excitability.

In conclusion, in healthy individuals, the simultaneous stimulations of MN and SON increased the excitability of the R1 circuit. In contrast, the R2 response magnitude was reduced, exhibiting a recovery curve similar to that of paired SON stimulation. Understanding the response characteristics of brainstem BR circuitry may allow us to understand further the pathophysiology of disorders which are direct results of pathology in the facial nucleus, such as hemifacial spasm or postfacial synkinesis, or the disorders that change the excitability of the brainstem such as dystonia, Huntington disease, or Parkinson’s disease.

## Figures and Tables

**Table 1:** Comparison of the single SON, single MN, and the simultaneous SON+MN stimulations.

Recording parameters	Single SON stimulation n=18	Single MN stimulation n=18	p*	SON+MN stimulation n=18	p**	p***
R1 latency, ms	10.2±0.6	12.0±1.6^a^	0.058	9.6±0.6	0.003^y^	0.028^y^
R1 amplitude, μV	340.2±310.2	88.6±53.0^a^	0.028^z^	491.9±345.0	0.007^z^	0.018^z^
R2 latency, ms	32.6±2.9	38.2±8.1	0.007^y^	30.4±1.9	0.000^y^	0.001^y^
R2 AUC, mV ms	2.7±1.5	1.9±1.3	0.037^z^	2.8±2.4	0.647	0.022^z^
R2c latency, ms	33.4±3.5	41.0±8.5	0.003^y^	30.9±2.1	0.008^y^	0.000^y^
R2c AUC, mV ms	1.7±0.7	1.1±0.7	0.002^z^	2.1±0.9	0.047^z^	0.005^z^

All values are given as mean ± SD. SON, supraorbital nerve; MN, mentalis nerve; AUC, area under-the-curve

* Comparison of SON and MN stimuli;

** Comparison of SON and SON + MN stimuli;

*** Comparison of MN and SON + MN stimuli.

a seven individuals had an R1 response after MN stimulation.

p values of R1 amplitude, R2 AUC, and R2c AUC were obtained by Wilcoxon signed-rank test.

p values of R1 latency, R2 latency, and R2c latency were obtained by repeated measures ANOVA.

z < 0.05,

y < 0.005

**Table 2:** Comparisons of the R1 amplitude and R2 AUCs of the simultaneous SON+MN stimulation and arithmetic sum after the single MN and single SON.

	Arithmetic sum n=18	Simultaneous SON+MN stimulations n=18	p
**R1 amplitude, μV**	317.9±306.3^a^	491.9±345.0	**0.009** z
**R2 AUC, mV ms**	4.5±2.5	2.8±2.4	**<0.001** y
**R2c AUC, mV ms**	2.5±1.5	2.1±0.9	**0.008** z

a Only seven individuals had an R1 response after MN stimulation.

Comparisons were done using the Wilcoxon signed-rank test.

z < 0.05,

y < 0.001;

SON, supraorbital nerve; MN mental nerve; AUC, area under-the-curve
